# Empowering patients with diabetic retinopathy

**Published:** 2015

**Authors:** Peter Blows, Tunde Peto, Katlego Mbulawa

**Affiliations:** Ophthalmic Image Grader: Moorfields Eye Hospital, London, UK; Ophthalmologist & Head of The Reading Centre: NIHR Biomedical Research Centre at Moorfields Eye Hospital NHS Foundation Trust and UCL Institute of Ophthalmology, London, UK; Ophthalmic Nurse: Botswana National Diabetic Eye Screening Service, Gaborone, Botswana

Knowing how to engage and enthuse patients with diabetes is crucial to preventing blindness due diabetic eye disease, especially taking rising prevalence into account. Whilst treatment methods and facilities vary across the world, the mantra of prevention and empowerment remains constant. This article includes contributions from both a UK and an African screening service to highlight ways to tackle similar challenges.

Whatever the setting, it is important to identify and understand the needs of your community. Tower Hamlets in London, for example, has the largest Bangladeshi population in England and both verbal and written information is available in Bengali.

Raising awareness is also important. In the Botswana National Programme, activities to raise awareness in the community and in the health care sector include road shows, TV and radio presentations, and the distribution of diabetes pamphlets and small pocket-size cards with helpful contact information. The programme also carries out patient workshops, cardiovascular disease education, diabetes education, consultations and retinal screening.

People with diabetes have many appointments to keep, including foot examinations and blood glucose, blood lipids and blood pressure tests. It is important for eye care professionals not assume that patient education has already been given elsewhere, and every opportunity must be taken to encourage patients to take good care of themselves (see ‘**Information for patients: living with diabetes and protecting your eyes**’).

Many complications of diabetes, including blindness, can be prevented if patients have the appropriate information and advice and feel empowered to take ownership of their condition. Signposting all services available to people with diabetes is therefore everybody's responsibility. Diabetic retinopathy (DR) is often symptomless until the late stages and this presents a real challenge to uptake of diabetic eye checks the world over.

Diabetes UK has developed a list of **15 essential checks and services** that people with diabetes should be receiving. DR screening services in England are encouraged to signpost these for their patients. If you work in diabetes care, would you know where to advise your patient to access the checks and services included in this list? Whilst some patients may already be aware of them, others might not have ever been informed and are therefore at risk of complications.

## Identification of diabetic retinopathy

Management of DR occurs in a variety of settings. Screening in the community (such as in the UK and many other countries) means that patients are only referred to an ophthalmologist when they have significant levels of retinopathy. This has greatly reduced the strain on hospital eye services and allows ophthalmologists to concentrate on those with sight-threatening DR. Elsewhere in the world, people with diabetes have their eyes monitored by their ophthalmologist. Moving a camera into the diabetic clinic is an effective first step to increase screening numbers.

## If your patient has been diagnosed with diabetic retinopathy:

Background DR is the earliest stage of damage to the retina and does not require a referral. These findings are common in screening and can be stabilised with management of diabetes, blood pressure and cholesterol.

It is important to remember that, although background DR is not itself immediately sight-threatening, the patient may be anxious about their vision and so reassurance, education and advice will help the patient to make good decisions, such as coming back for screening and treatment (if needed), and managing their blood glucose and lipid levels, all of which can prevent sight loss from DR. Providing sound advice to patients at this stage is key to empowerment and prevention.

**Questions that may arise from a diagnosis of background DR.**

**Am I going to go blind?**

With good management of your condition, these changes may have stabilised by your next visit with no disturbance to your sight.

**I have no symptoms, so do I still need to come back next year?**

Yes, whilst these changes are not yet sight threatening, seeing you again will ensure that any progression of your condition is picked up early, before you notice symptoms.

Pre-proliferative DR, proliferative DR and maculopathy are potentially sight threatening, and patients may present with reduced vision. These patients require further assessment by an ophthalmologist. Effective communication in hospital eye clinics is essential. At Moorfields, Eye Hospital, those who attend the eye clinic will have further tests so the best possible treatment can be offered, whether laser treatment, injections, surgery or simply close monitoring. The most important thing is that you discuss all the patient's questions with them. All treatments require regular attendance at the eye clinic and so it is important that the patient can attend all appointments (and understands why this is important for them). Not coming back to the clinic can mean that the eye disease can progress quickly to the point where their sight might be lost.

## Key points to remember for patients and health care professionals

- DR is symptomless in its early stages and early identification is vital
- DR can cause blindness if it is not recognised and treated early
- Effective treatment **is** available that will almost always prevent sight loss **if** eye disease is identified early
- DR screening is not a complete eye test and is undertaken *alongside* an annual optician review
- Self-care advice is the same as for other complications of diabetes: well controlled blood glucose and blood pressure, a healthy diet and regular exercise
- Patients must attend their appointments as these are valuable opportunities for discussion and treatment.

With education, support and understanding we can help to ensure that our patients are in control of their condition and know how to prevent further complications. Nobody **wants** to go blind and this approach can go a long way to preventing sight loss due to diabetes.
